# Adaptive Probabilistic RREQ Rebroadcasting Using Thompson Sampling for Mobile Ad Hoc Sensor Networks

**DOI:** 10.3390/s26154870

**Published:** 2026-08-02

**Authors:** Dimitra G. Kampitaki, Anastasios A. Economides

**Affiliations:** Information Systems IPSS, University of Macedonia, 54636 Thessaloniki, Greece; economid@uom.gr

**Keywords:** mobile ad hoc sensor networks, AODV, RREQ suppression, broadcast storm, Thompson sampling, adaptive routing, multi-armed bandit

## Abstract

**Highlights:**

**What are the main findings?**

- A lightweight Thompson-sampling mechanism is proposed to adapt the RREQ rebroadcast probability in AODV-based mobile ad hoc sensor networks.
- The proposed method reduces normalised routing overhead and RREQ forwarding compared with fixed probabilistic rebroadcasting, while no statistically significant PDR difference was observed under the tested conditions.

**What are the implications of the main findings?**

- Fixed probabilistic RREQ suppression can be improved through lightweight online probability selection rather than by using a single manually selected forwarding probability.
- The method requires no additional control packets, topology exchange, or packet format modification. Its persistent learning state contains 30 local Beta parameters per node, supplemented by temporary pending-decision records.

**Abstract:**

Route discovery in ad hoc on-demand distance vector (AODV)-based mobile ad hoc sensor networks relies on route request (RREQ) dissemination, which improves reachability, but generates redundant rebroadcasts, channel contention, delay, and energy waste. Fixed probabilistic rebroadcasting mitigates broadcast storms, but its performance depends on a manually selected forwarding probability applied uniformly across different local redundancy conditions. This work proposes the Thompson-sampling probabilistic AODV (TSP-AODV), a lightweight adaptive extension, in which each intermediate node selects among a small set of forwarding probability arms using a local duplicate-pressure context and delayed route reply (RREP) feedback. The reward function uses the local observation of a corresponding RREP as delayed feedback while penalising high forwarding probability in locally redundant contexts. TSP-AODV requires no additional control packets, no topology exchange, and only a small number of local Beta belief distribution parameters per node. Evaluated against AODV, fixed probabilistic rebroadcasting, counter-based suppression, and dynamic probabilistic-counter suppression over 1600 simulation runs spanning four node densities and four mobility levels, TSP-AODV achieves the lowest normalised routing overhead and the highest RREQ suppression ratio, while no statistically significant PDR difference relative to the fixed probabilistic baseline was observed under the tested conditions. End-to-end delay is also reduced significantly relative to fixed probabilistic rebroadcasting. The learned belief behaviour confirms context-dependent adaptation, with the dominant forwarding-probability arm decreasing as duplicate pressure increases. An additional 800-run sensitivity analysis characterises the PDR–NRO–delay trade-off across the tested penalty coefficients and feedback-window durations in two representative scenarios. These results are limited to the evaluated parameter grid and do not establish scenario-independent parameter robustness.

## 1. Introduction

Mobile ad hoc sensor and Internet of Things (IoT) networks require routing mechanisms capable of delivering data over dynamic, multi-hop wireless topologies without fixed infrastructure. Application domains include environmental monitoring, disaster response, industrial sensing, vehicular sensing, tactical communications, and temporary smart-city deployments [1]. Routing overhead directly affects energy consumption, channel availability, delay, and sensing-data delivery, so route discovery must remain reliable while minimizing unnecessary control traffic.

Ad hoc on-demand distance vector (AODV) [2] is a widely deployed reactive routing protocol. When a source node requires a route to a destination, it broadcasts a route request (RREQ). Intermediate nodes rebroadcast fresh RREQs until the request reaches the destination or a node holding a valid route, at which point a route reply (RREP) is unicast back to the source. While effective, this mechanism generates redundant rebroadcasts, channel contention, packet collisions, delay, and energy waste, which is the classic broadcast storm problem [3].

Broadcast storm mitigation approaches include probabilistic, counter-based, distance-based, location-based, cluster-based, and neighbour-knowledge-based methods [3,4]. Probabilistic rebroadcasting forwards each fresh RREQ with a fixed probability p. Counter-based schemes suppress rebroadcasts once enough duplicate copies have been observed. Dynamic probabilistic-counter schemes map the duplicate count to a decreasing forwarding probability. All these methods depend on statically chosen parameters whose optimality varies with network density, mobility, and route-discovery fragility, conditions that change continuously in realistic mobile sensor deployments.

Recent AODV studies continue to pursue adaptive route-discovery and routing mechanisms, including probabilistic RREQ forwarding [5], highly mobile aerial-network routing [6], energy-aware route weighting [7], cross-layer fuzzy routing [8], and resilience-oriented MANET routing [9]. However, learning-based and bandit-based routing studies mainly address route, next-hop, path, or forwarding-candidate selection [10,11,12], rather than the specific problem of learning the RREQ rebroadcast probability itself. This gap motivates the proposed TSP-AODV mechanism.

This work revisits probabilistic RREQ suppression as an online forwarding-probability selection problem. The proposed Thompson sampling probabilistic AODV (TSP-AODV) mechanism keeps the structural simplicity of probabilistic rebroadcasting but replaces the fixed forwarding probability with Thompson sampling [13], which is a Bayesian bandit algorithm. Each node maintains Beta belief distributions over a small set of forwarding-probability arms, indexed by a local duplicate-pressure context derived from duplicate RREQ observations. A reward signal is generated when the node locally observes a corresponding RREP within the feedback window. This observation serves as a proxy for the usefulness of the selected forwarding-probability arm while penalising high forwarding probability under strong redundancy evidence. The method introduces no additional control packets and stores only a small number of scalar Beta belief parameters per node. The contribution is a lightweight online mechanism for adapting the intermediate-node RREQ rebroadcast probability during AODV route discovery.

The main contributions of this work are as follows:

1. Probabilistic RREQ rebroadcasting in AODV is formulated as a local contextual bandit problem, where duplicate pressure defines the local context and forwarding probability is the selected action.
2. TSP-AODV introduces delayed RREP-based feedback and a redundancy-aware bounded reward that reinforces useful route-discovery participation while penalising high forwarding probability in redundant contexts.
3. The mechanism requires no additional control packets, no topology exchange, and no packet-format modification, and is evaluated against AODV, fixed probabilistic, counter-based, and dynamic probabilistic-counter baselines, including sensitivity analysis.

## 2. Related Work

Tseng et al. [3] introduced the broadcast storm problem, demonstrating that uncontrolled rebroadcasting causes redundancy, contention, and collision in mobile ad hoc networks. Williams and Camp [4] compared broadcasting techniques across probabilistic, counter-based, distance-based, location-based, cluster-based, and neighbour-knowledge-based families, establishing the classical taxonomy of broadcasting suppression methods. Haas, Halpern, and Li [14] showed through gossip-based ad hoc routing that routing overhead can be substantially reduced while preserving high dissemination coverage, provided that the forwarding probability is chosen appropriately. More recent surveys of probabilistic broadcast schemes confirm that such methods can reduce overhead in wireless ad hoc networks but also emphasise their sensitivity to forwarding probability selection and network conditions [15]. The core limitation of probabilistic methods is parameter sensitivity: a single fixed probability is applied under all local redundancy, density, and mobility conditions.

Adaptive routing research in MANETs and AODV spans several protocol functions beyond RREQ rebroadcasting. Hoang et al. improve fault recovery in on-demand routing [16], Chandravanshi et al. address energy- and bandwidth-aware multipath load balancing [17], and Kaddoura et al. adapt on-demand route construction to changing network conditions [18]. Binh and Duong use reinforcement learning for QoS-aware route selection [19], Li et al. incorporate local neighbour density into route selection [20], and Ozen and Ozen prioritise emergency traffic within the routing process [21]. Other approaches combine adaptive expanding-ring search and random early detection with machine learning classifiers [22], use fuzzy reinforcement learning to control broadcast decisions [23], multi-criteria relay selection [24], learning-assisted reduction in routing-update costs [25], clustered hardware-assisted AODV operation [26], or reinforcement learning with swarm optimisation for secure path selection [27]. Among these studies, the broadcast-control mechanism in [23] is closest to the intervention point considered here, but it does not learn a context-indexed RREQ forwarding-probability arm through Thompson sampling.

More directly relevant to TSP-AODV, Shete and Awale [28] proposed a channel-quality-based adaptive gossip mechanism for AODV, adjusting forwarding behaviour according to wireless-link conditions. Cai and Qian [5] proposed an AODV variant based on probabilistic flooding and multi-constraint fusion, where RREQ forwarding probability is computed using multiple local criteria. Other adaptive AODV variants operate at broader routing or protocol levels. Tu [6] proposed SHARP-AODV, an intelligent adaptive AODV variant for highly mobile autonomous aerial vehicle networks, using local network and link-quality indicators to improve routing decisions. Other AODV extensions use energy-aware, fuzzy, cross-layer, or resilience-oriented mechanisms to improve route selection and protocol robustness [7,8,9]. These approaches demonstrate that adaptive AODV design remains relevant, but most of them rely on predefined scoring rules, engineered weighting functions, fuzzy inference, or protocol-specific heuristics. They do not learn the RREQ forwarding probability from local duplicate-pressure context and delayed RREP feedback.

Counter-based schemes introduce local redundancy awareness but require manual threshold selection. A node waits for a short interval, counts duplicate copies of the same broadcast packet, and suppresses rebroadcasting if the duplicate count exceeds a threshold [3,4]. Dynamic probabilistic-counter schemes soften this decision by converting duplicate count into a forwarding probability, usually through a predefined decreasing function. These mechanisms are adaptive in form because their decision changes with duplicate evidence, but the threshold or adaptation curve is fixed before deployment. Therefore, their performance can still be sensitive to density, mobility, and traffic conditions. 

Mobile sensor and IoT routing studies provide a closer resource-constrained context. Temene et al. survey how node and sink mobility changes connectivity, data collection, and routing requirements in wireless sensor networks [29]. Al-Sadoon et al. use dual-tier clustering for mobile sensor routing [30], while Godfrey et al. [31] and Alsalmi et al. [32] employ reinforcement learning to improve energy-aware routing in software-defined or mobile wireless sensor networks. Ghori et al. [33] propose a multipath-optimised AODV variant for BLE mesh networks to improve reliability and route recovery under node failures. These methods address mobility management, clustering, energy-aware path selection, or controlled route discovery. None of them learns to select among local RREQ rebroadcast-probability arms using delayed RREP feedback.

High-mobility networking studies provide complementary evidence on the use of online learning under rapidly changing topologies. Cui et al. use adaptive Q-learning for topology-aware FANET route selection [34], Jiang et al. develop environment-aware reinforcement-learning routing for VANETs [35], and Sun et al. combine Q-learning with AODV multipath selection in FANETs [36]. These studies show that online learning can respond to rapid topology change. Their action spaces, mobility assumptions, and protocol intervention points differ from TSP-AODV, so they are treated as adjacent literature and not as direct RREQ-suppression predecessors or experimental baselines.

Adaptive suppression has also been studied for low-power wireless protocols: the Trickle algorithm [37] suppresses transmissions based on consistency and local redundancy, whereas Meyfroyt et al. [38] and Coladon et al. [39] showed that adaptive or multiple redundancy constants can improve performance under heterogeneous conditions. These works are not AODV route-discovery mechanisms, but they reinforce the broader observation that static suppression parameters are often suboptimal in wireless networks with changing local redundancy.

Learning-based routing has attracted growing attention, with Q-learning, deep reinforcement learning, and bandit methods applied to routing-table optimisation, next-hop selection, channel selection, and rate adaptation. Recent surveys show that reinforcement-learning-based routing can improve adaptation in vehicular and mobile ad hoc environments, but such methods often address broader routing optimisation problems and may require richer state representations, longer convergence periods, or heavier protocol modifications [10]. Multi-armed and contextual bandit methods provide a lighter alternative when the decision-maker must select among a finite set of actions using limited feedback [11].

Recent comparative evaluations under different traffic loads [40] and dynamic network conditions [41] also caution against treating one routing configuration as universally best. This supports the multi-density and multi-mobility evaluation used here and the restricted interpretation of the two-scenario sensitivity study. TSP-AODV nevertheless has a narrower intervention point, as it preserves the AODV route-selection process and changes only the probabilistic decision to rebroadcast a fresh RREQ.

Thompson sampling [13] is a Bayesian bandit method that naturally balances exploration and exploitation. In the Beta-Bernoulli setting, each arm maintains a Beta belief distribution. At each decision epoch, one value is sampled from each belief, the arm with the highest sampled value is selected, and only the selected arm is updated after feedback is observed. This makes Thompson sampling attractive for resource-constrained environments. Although Thompson sampling and related bandit methods have been applied to networking and routing problems, including route, next-hop, and opportunistic forwarding selection [11,12], their use has mainly targeted the selection of data-forwarding paths or forwarding candidates. To the best of our knowledge, Thompson sampling has not been explicitly formulated for the narrower AODV route-discovery problem of selecting the RREQ rebroadcast probability itself. 

In this work, TSP-AODV applies this principle to the specific and narrow problem of adaptive RREQ forwarding-probability selection. Unlike broader learning-based routing approaches that optimise routes or next-hop choices, TSP-AODV modifies only the intermediate-node RREQ rebroadcast decision. Each node learns among a small set of forwarding-probability arms using duplicate-pressure context and delayed RREP feedback. The method therefore preserves the lightweight structure of probabilistic rebroadcasting while allowing the forwarding policy to adapt online instead of relying on a single fixed probability or a predefined duplicate-count curve.

Contextual-bandit AODV variants that learn route, next-hop, or path-selection policies are not directly equivalent baselines because their action space and protocol intervention point differ from TSP-AODV. TSP-AODV acts only at the intermediate-node RREQ rebroadcast decision and deliberately preserves the original AODV route-selection process. Therefore, comparing TSP-AODV with route-selection or next-hop-selection bandit schemes would mix different protocol intervention points; such schemes are discussed as related learning-based routing approaches, whereas the experimental baselines are restricted to mechanisms that modify the RREQ rebroadcast/suppression decision.

Table 1 is limited to works that directly define the broadcast-suppression, adaptive RREQ-forwarding, and Thompson-sampling design space considered in this study. Broader studies that optimise routes, paths, relays, clustering, routing updates, or protocol configuration are discussed in the text but are not included as direct method-level comparisons.

## 3. Proposed TSP-AODV

### 3.1. RREQ Suppression Model

Consider a mobile ad hoc sensor network with node set $N\;=\;\{n_{1},\;n_{2},\;\ldots ,{\;n}_{N}\}$ and time-varying topology graph G(t) = (N, E(t)). When a source has no valid route to a destination, it initiates route discovery by broadcasting an RREQ q (identified by originator address and RREQ ID). When an intermediate node nᵢ receives a fresh RREQ q, it must decide whether to forward or suppress it, aᵢ(q) ∈ {forward, suppress}. Standard AODV forwards fresh RREQs according to the AODV route discovery process [2], whereas fixed probabilistic AODV forwards with static probability p, following the probabilistic broadcast-suppression principle used in prior broadcast-storm mitigation studies [3,4,14]. TSP-AODV selects p online from a finite action set. 

### 3.2. Motivation for Context-Dependent Probability Selection

A fixed forwarding probability must serve both low-redundancy situations, where suppression may prevent route discovery from progressing, and high-redundancy situations, where another rebroadcast is more likely to be unnecessary. We denote s∈S a discrete duplicate-pressure context and p∈A a candidate forwarding probability. The duplicate pressure score $z_{i}\left(q\right)$ is continuous in [0, 1] and may vary across RREQs even within the same discrete context. Therefore, the utility of probability p in context s is defined as the context-conditional expected reward:

$$U\left(s,p\right)=E_{z|s}\left[\rho \left(z,p\right)\left(1-\lambda pz\right)\right].$$

Here, $\rho \left(z,p\right)$ is the route-discovery success probability under duplicate-pressure score z and forwarding probability p, and λ controls the penalty assigned to high forwarding probability under redundant conditions. 

A fixed probabilistic scheme selects one probability for all contexts:

$$p_{fixed}^{*}=arg\underset{p\in A}{max}E_{s}\left[U\left(s,p\right)\right].$$

A context-dependent policy can instead select a different probability in each context:

$$p^{*}\left(s\right)=arg\underset{p\in A}{max}U\left(s,p\right).$$

The best context-dependent policy is at least as expressive as the best fixed-probability policy:

$$E_{s}\left[\underset{p\in A}{max}U\left(s,p\right)\right]\geq \underset{p\in A}{max}E_{s}\left[U\left(s,p\right)\right].$$

This follows because ${max}_{p}U\left(s,p\right)\geq U\left(s,p^{*}\right)$ for any fixed $p^{*}$ and any context s, since taking expectations preserves the inequality. The inequality does not imply that the learned policy will always outperform every baseline in finite simulations. It only shows that a single fixed forwarding probability is structurally limited when the best probability differs across local redundancy conditions.

### 3.3. Duplicate-Pressure Context

When node nᵢ receives a fresh RREQ q, it waits for a short assessment interval $\Delta_{assess}$ and counts duplicate copies of the same RREQ. Let Dᵢ(q) be the number of duplicates observed during this interval. The normalised duplicate-pressure score is:

$$zᵢ(q)\;=\;min\left(\frac{Dᵢ(q)\;}{D_{ref}},\;1\right)$$

where $D_{ref}$ is a reference duplicate count, which for the evaluated configuration was set to $D_{ref}$ = 3. This duplicate-counting principle follows the counter-based suppression rationale originally used to reduce redundant rebroadcasts in broadcast-storm mitigation [3,4].

The assessment interval introduces a small intentional delay before forwarding an intermediate-node RREQ. This delay is the price paid for observing local duplicate pressure before deciding whether another rebroadcast is useful. The feedback window used for learning does not delay the RREQ itself but only determines how long the node keeps the pending decision record before assigning delayed feedback. Therefore, the forwarding-delay cost is associated with the assessment interval, whereas the reward window affects learning accuracy and credit assignment. This creates a deliberate trade-off: a short local waiting time improves duplicate-pressure estimation, but it can also increase route-discovery delay in cases where the additional redundancy information does not compensate for the waiting time.

The normalised score is mapped to a discrete context sᵢ(q) ∈ S, where S = {low, medium, high}, according to

$$sᵢ\left(q\right)=\left\{\begin{array}{c} low,\;\;z_{i}(q)=0,\\ medium,\;\;0<z_{i}\left(q\right)<1,\\ high,\;\;z_{i}(q)=1. \end{array}\right.$$

Given $D_{ref}=3$, these contexts correspond to $Dᵢ\left(q\right)=0,$ $Dᵢ\left(q\right)\in \left\{1,2\right\},$ and Dᵢ(q)≥3, respectively. Duplicate counts equal to or greater than $D_{ref}$ are clipped to $z_{i}(q)=1$ and assigned to the high-pressure context. These boundaries are applied identically in all principal and sensitivity-analysis runs. The context definition is intentionally simple and requires no neighbour tables, topology estimation, location information, or additional control packets.

### 3.4. Probability Arms and Thompson Sampling

Following the Thompson-sampling principle of sampling from arm-specific belief distributions before selecting an action [13], each node selects a forwarding probability from a finite set, $A\;=\;\{p_{1},\;p_{2},\;\ldots ,{\;p}_{K}\}$, which in this work is set to A = {0.5, 0.6, 0.7, 0.8, 0.9}. For each context sᵢ and probability arm $p_{k},$ node $n_{i}$ maintains two Beta belief parameters, $\alpha_{i,s,k}$ and $\beta_{i,s,k}$ initialised as $\alpha_{0}=\beta_{0}=1.$ These parameters are initialised independently at every node at the beginning of each simulation run. No learned belief state is transferred across repetitions, density–mobility scenarios, protocol variants, or sensitivity-analysis settings. When an RREQ q is assigned to context sᵢ(q), node $n_{i}$ samples $\theta_{i,s,k}\;\sim \;Beta(\alpha_{i,s,k},\;\beta_{i,s,k})$ for every candidate arm $p_{k}.$ The selected arm is $k^{*}\;=\;arg\;{\underset{k}{\max}\theta }_{i.s.k},$ and the selected forwarding probability is $p^{*}\;$= $p_{k^{*}}$. The node then draws u uniformly from [0, 1]. If $u\;\leq \;p^{*}$, the RREQ is forwarded, otherwise, it is suppressed.

### 3.5. Delayed RREP Feedback and Reward

The effect of an RREQ forwarding probability arm cannot be evaluated immediately. After TSP-AODV selects arm $p^{*}\;$and realises either a forwarding or suppression action, the node stores a pending decision record containing the RREQ identifier, originator address, destination address, selected context, selected arm, selected forwarding probability, duplicate-pressure score, realised action, and expiry time. When an RREP is received, positive feedback is assigned to each unexpired pending record having the same originator and destination addresses. The RREQ identifier is retained in the pending record for identification and diagnostics, but it is not used for feedback matching because the standard AODV RREP does not carry the corresponding RREQ ID.

Because feedback matching uses only the originator–destination pair, one received RREP may update multiple unexpired pending records associated with that pair. If route discoveries for the same originator and destination overlap within the feedback window, positive feedback may be assigned to a decision that did not causally contribute to the observed RREP. The finite feedback window limits the temporal extent of this ambiguity but does not eliminate it.

Let $I_{RREP}\left(q\right)=1$ when such a source–destination-matching RREP is observed within the reward window and let $I_{RREP}\left(q\right)=0$ otherwise. The feedback indicates that the node observed a corresponding RREP within the reward window. It does not directly establish whether global route discovery succeeded through another branch. The reward assigned to the selected context-arm pair is

$$R_{i}\left(q,p^{*}\right)=I_{RREP}\left(q\right)\left[1-\lambda p^{*}z_{i}\left(q\right)\right].$$

In the evaluated configuration,

λ=0.35.

The coefficient λ∈[0, 1] controls the strength of the redundancy penalty. When λ=0, successful route discovery receives the same reward regardless of the selected forwarding probability and observed duplicate pressure. As λ increases, successful decisions using high forwarding probabilities under strong duplicate pressure receive progressively lower rewards. Therefore, smaller values favour route-discovery reliability, whereas larger values encourage stronger RREQ suppression. Restricting λ to [0, 1] keeps the reward bounded because $p_{k}$, $z_{t}\in [0,\;1]$.

The value λ=0.35 was initially selected as a moderate operating point and not obtained through optimisation. At this value, the route-discovery feedback term remains dominant, while the redundancy penalty is strong enough to distinguish high-probability rebroadcasting under dense local RREQ dissemination. For example, when $z_{i}(q)=1$ and $p^{*}=0.9$, a successful decision receives 1−0.35⋅0.9=0.685, whereas under low duplicate pressure $z_{i}(q)\approx 0$, the reward remains close to one. This makes λ=0.35 a conservative compromise between preserving route-discovery reliability and discouraging redundant high-probability rebroadcasting. This value is not universally optimal, and its effect is evaluated through the sensitivity analysis in Section 4.5 and Section 5.8 using values below and above the selected configuration.

Failed or expired decisions receive zero reward. Successful route-discovery feedback receives a reward close to one under low duplicate pressure, while under high duplicate pressure the reward is reduced more strongly for higher forwarding probabilities. This reward structure favours successful route discovery while discouraging unnecessary high probability rebroadcasting in redundant contexts.

The pending record is stored for every TSP-AODV probability-arm decision, not only for forwarded RREQs, meaning the number of reward updates follows the number of completed TS decisions rather than the number of forwarded RREQs. This is consistent with the purpose of the learner: it evaluates the selected probability arm under the observed duplicate-pressure context, while the actual forward/suppress action is generated probabilistically from that selected arm. Suppression is learned indirectly through the selection of lower-probability arms, not through a separate suppression-specific reward.

### 3.6. Statistical Interpretation of the Fractional Beta Update

The reward $R_{i}\left(q,p^{*}\right)$ is bounded in $\left[0,1\right]$, but it is not a Bernoulli observation. A successful route discovery feedback event may receive a fractional value lower than one when the selected forwarding probability is high under strong duplicate pressure. As a result, the update used in TSP-AODV is not an exact Beta-Bernoulli conjugate posterior update. Instead, the Beta variables are used as compact bounded-utility belief parameters for each context-arm pair. The update

$$\alpha_{i,s,k^{*}}\leftarrow \alpha_{i,s,k^{*}}+R_{i}\left(q,p^{*}\right),$$

$$\beta_{i,s,k^{*}}\leftarrow \beta_{i,s,k^{*}}+1-R_{i}\left(q,p^{*}\right),$$

treats each completed decision as one bounded-feedback observation whose positive evidence is $R_{i}\left(q,p^{*}\right)$ and whose complementary evidence is $1-R_{i}\left(q,p^{*}\right)$. Thus, $\frac{\alpha }{\alpha +\beta }$ is used as a smoothed estimate of bounded utility, not as a literal Bernoulli success probability. This maintains the lightweight Thompson-sampling selection rule while allowing the delayed RREP feedback to encode both route-discovery usefulness and redundancy cost. Similar Thompson-sampling formulations for bounded reward distributions are considered in [42]. Accordingly, TSP-AODV is best understood as a Thompson-sampling-style bounded-feedback heuristic and not an exact Bayesian posterior sampler under a Bernoulli likelihood.

The complete TSP-AODV decision and update procedure is summarised in Algorithm 1. The algorithm stores a pending record for every selected probability-arm decision, whether the realised local action is forwarding or suppression. The selected context-arm pair is then updated using delayed RREP feedback or expiry of the reward window. The update in Lines 23–24 is a fractional pseudo-count update for bounded utility feedback. It preserves the Thompson-sampling sampling-and-selection mechanism but does not claim exact Bernoulli conjugacy.
**Algorithm 1.** TSP-AODV RREQ rebroadcast decision and delayed update**Input:** fresh RREQ q, node $n_{i}$, previous-hop address, TTL**State:** Beta belief parameters $\alpha_{i,s,k}$ and $\beta_{i,s,k}$ for all $s_{i}$ in S and $p_{k}$ in A
**Output:** forward or suppress decision; updated belief parameters*Initialisation, performed once per node at the beginning of each simulation run:*1:     A ← {0.5, 0.6, 0.7, 0.8, 0.9};    S←{low, medium, high}
2:     $D_{ref}\;\leftarrow \;3$;    λ ← 0.35;    $\alpha_{i,s,k}\;\leftarrow \;1$;    $\beta_{i,s,k}\;\leftarrow \;1$
*Decision process:*3:     wait $\Delta_{assess\;}$ and count duplicate copies D of q
4:     $z\;\leftarrow \;min((D\;)/D_{ref}\;,\;1)$
5:     s ← map(z)  to {low, medium, high}
6:     **for** each arm k in A do7:         $\theta_{k}$ ← sample Beta($\alpha_{i,s,k}$, $\beta_{i,s,k}$)8:     **end for**9:     $k^{*}\leftarrow \;argmax$ over k of $\theta_{k}$;    $p^{*}\leftarrow A[k^{*}]$
10:   u ← Uniform(0,1)
11:   **if** $u\;\leq \;p^{*}$ then12:       forward q
13:   **else**14:       suppress q
15:   **end if**16:   store pending record (q.id, q.originator, q.destination, s, $k^{*}$, $p^{*}\;$, z, action, expiry)*Reward and belief update:*17:   **if** matching RREP(q) is observed before expiry then18:       $I_{RREP}\;\leftarrow \;1$
19:   **else**20:       $I_{RREP}\;\leftarrow \;0$
21:   **end if**22:   $R\;\leftarrow \;I_{RREP}\;\cdot \;(1\;-\;\lambda \;\cdot \;p^{*}\;\cdot \;z)$
23:   $\alpha_{i,s,k}$_*_ ← $\alpha_{i,s,k}$_*_ + R
24:   $\beta_{i,s,k}$_*_ ← $\beta_{i,s,k}$_*_ + 1 − R
25:   remove pending record

### 3.7. Complexity

With |S| contexts and K probability arms, each node stores: 2 · |S| · K persistent Beta belief parameters. In the evaluated implementation $\left|S\right|=\;3,\;\;K\;=\;5,$ so each node stores 2 · 3 · 5 = 30 scalar belief parameters. Sampling the arm-specific Beta distributions and selecting the arm with the largest sampled value require $O\left(K\right)$ operations per RREQ decision.

The delayed-feedback mechanism additionally requires temporary pending-decision records. Let $M_{i}(t)$ denote the number of unexpired records stored by node $n_{i}$ at time t. Each record has constant size and stores the information required to identify the decision, assign delayed feedback, and remove the record after feedback or expiry. The node-level storage requirement is $O\left(\left|S\right|\cdot K+M_{i}(t)\right).$ The value of $M_{i}\left(t\right)$ depends on the local rate of RREQ decisions and the feedback-window duration W. Because each record is removed when matching feedback is received or when its feedback window expires, $M_{i}(t)$ is bounded by the number of decisions made locally during the preceding W-second interval. In the current sequential record representation, inserting a record requires constant time, whereas feedback matching and expiry processing may require examining up to $M_{i}(t)$ records. Their worst-case processing cost is $O\left(M_{i}(t)\right)$. The simulations did not record the time-averaged or maximum pending-record occupancy, so empirical typical and maximum values cannot be reconstructed from the available scalar outputs. The incremental communication overhead is zero because no additional control packets are transmitted, and no packet fields are modified.

## 4. Evaluation Methodology

### 4.1. Simulation Setup

The proposed method was evaluated using OMNeT++ 6.3.0 with the INET 4.5.4 framework. All variants use identical network, mobility, traffic, MAC, and radio settings, differing only in the RREQ rebroadcast decision mechanism. Table 2 describes the five compared variants and Table 3 lists the simulation parameters.

For DynProbCounter, the forwarding probability after observing D duplicate RREQs is

$$p_{DPC}\left(D\right)={clip}_{\left[0,1\right]}\left[p_{min}+\left(p_{max}-p_{min}\right)\cdot e^{-\delta \frac{D}{D_{ref}}}\right],$$

where $p_{min}=0.35$, $p_{max}=1.0,$ δ=1.0, and $D_{ref}=3.$ At D=0, the forwarding probability is $p_{DPC}\left(0\right)=1.$ It decreases monotonically as the duplicate count increases and approaches, but does not fall below, $p_{min}=0.35$. The node rebroadcasts the RREQ when a uniformly distributed random value u∈[0,1] satisfies $u\leq p_{DPC}\left(D\right)$, and suppresses it otherwise.

The network operates in a 1000 m × 1000 m area under random waypoint mobility. Four node densities (N ∈ {20, 30, 40, 50}) and four speeds ($V_{max}\;\in \;\{2,\;5,\;10,\;20\}\;m/s$) were evaluated. The speed of each movement was sampled uniformly between 0.1 m/s and the scenario specific maximum speed $V_{max}.$ The pause time after reaching each destination was fixed at 2 s. Each combination was repeated 20 times with different random seeds: 5 × 4 × 4 × 20 = 1600 principal comparative runs. The targeted sensitivity analysis introduced an additional 800 TSP-AODV runs, as described in Section 4.5.

### 4.2. Traffic and MAC Configuration

Ten UDP source–destination pairs were configured using constant bit rate (CBR) traffic. Each source generated 512-byte UDP packets every 0.25 s, corresponding to 4 packets/s per flow and 40 packets/s in aggregate across the 10 active flows. The simulation duration was 125 s, with a 5 s warm-up period excluded from scalar metric collection. The wireless interface used the INET IEEE 802.11 MAC in ad hoc mode with an IEEE 802.11 scalar radio model, a 2 Mbps data rate, and a nominal communication range of approximately 250 m. All compared variants used identical MAC, radio, mobility, and traffic settings; they differed only in the RREQ rebroadcast decision mechanism. The evaluation focuses on routing-level metrics, namely PDR, end-to-end delay, normalised routing overhead, RREQ forwarding, and suppression ratio. Only scalar outputs were used in the present evaluation; therefore, within-run convergence trajectories are not reported.

### 4.3. Metrics

The metrics used are:

1. Packet delivery ratio (PDR):

$$PDR=\frac{received\;application\;packets}{sent\;application\;packets}\;$$

PDR is reported as a ratio in the tables and text, whereas figures display the same values as percentages for readability.

2. End-to-end delay (E2ED): average time between application-packet generation and reception, reported in seconds.
3. Normalised routing overhead (NRO):

$$NRO=\frac{transmitted\;AODV\;control\;packets}{received\;application\;packets}\;\;$$

4. RREQ forwarded count: number of RREQ rebroadcasts performed by intermediate nodes.
5. Global suppression ratio:

$$suppression\;ratio=\frac{suppressed\;fresh\;RREQs}{received\;fresh\;RREQs}\;\;$$

6. For TSP-AODV, the TS decision suppression rate is also recorded as the number of TSP suppress decisions divided by the total number of TSP probability-arm decisions. This internal metric differs from the global suppression ratio, which uses received fresh RREQs as the denominator and is comparable across all variants.
7. For TSP-AODV, final belief means are also recorded for each context-arm pair to evaluate whether the method learns interpretable probability preferences.

### 4.4. Statistical Analysis

Variants were compared using matched runs with the same density, mobility level, and repetition index. Unless otherwise stated, statistical significance was evaluated using a two-sided paired *t*-test, with p<0.05 considered significant. The primary comparison is TSP-AODV versus fixed probabilistic AODV because the proposed method is designed as an adaptive extension of fixed probabilistic rebroadcasting. Dynamic probabilistic-counter suppression serves as the strongest rule-based adaptive baseline. Scenario-level trends are reported in Figures 2–6 and confidence intervals are shown where included in the corresponding figures.

In addition to the pre-specified pooled comparison, scenario-level paired tests were performed separately for each N×Vmax condition using the 20 matched repetitions available for that condition. Because Table 6 contains 80 secondary tests (five metrics across 16 density–mobility conditions), the reported scenario-level *p*-values are exploratory and are not adjusted for multiplicity. No confirmatory inference is drawn from an isolated scenario-level *p*-value. The pooled matched comparison between TSP-AODV and fixed probabilistic AODV, reported in Table 5, remains the primary inferential analysis.

### 4.5. Sensitivity Analysis of the Redundancy Penalty Coefficient and Feedback Window

A targeted factorial sensitivity analysis was conducted to examine the effect of the redundancy-penalty coefficient λ, and the delayed-feedback window W. The tested values were:

λ∈{0, 0.20, 0.35, 0.50, 0.70}

and

$$W\in \left\{2,\;4,\;6,\;8\right\}\;s.$$

The analysis was performed under two representative operating conditions. The sparse, low-mobility scenario used N=20 and $V_{max}=2\;m/s$ and represents the condition in which route discovery is comparatively fragile. The dense, high-mobility scenario used N=50 and $V_{max}=20\;m/s$ and represents a demanding condition with extensive RREQ dissemination and rapidly changing connectivity. Each parameter combination was evaluated using 20 matched repetition seeds, resulting in 800 additional TSP-AODV runs.

All parameters other than λ and W were kept identical to the principal evaluation. The assessment interval $\Delta_{assess}\;$was kept fixed at uniform (0 ms, 10 ms) in all sensitivity runs. The analysis considered PDR, NRO, E2ED, RREQ forwarding, suppression ratio, and the proportion of completed decisions receiving positive RREP feedback. The objective was to characterise the effects of λ and W and determine how the selected configuration compares with alternative parameter combinations within the two evaluated scenarios.

### 4.6. Scope and Experimental Boundaries

The evaluation focuses on cooperative AODV-based mobile ad hoc sensor networks and isolates the effect of the RREQ rebroadcast decision. Selfish or malicious nodes are therefore not considered. The proposed learner uses only duplicate-pressure context, and additional features such as channel load, residual battery level, neighbour stability, or link-quality indicators could improve adaptation, but would also increase state complexity and implementation overhead.

The mobility evaluation uses random waypoint to support controlled comparison with established MANET/AODV simulation studies. Although the topology changes continuously during each run due to node mobility, the density and maximum-speed settings remain fixed within each scenario. Segmented nonstationary scenarios with abrupt changes in speed, density, or traffic load are not considered. Random waypoint may also concentrate nodes toward the centre of the simulation area and does not represent all realistic mobility patterns. Trace-driven or map-constrained mobility validation is therefore left for future work.

The learning process uses cumulative Beta updates and scalar run-level outputs. As a result, within-run learning trajectories and convergence-speed curves are not reported. In strongly nonstationary environments, selective belief forgetting or sliding-window updates may be needed to improve adaptation, but these mechanisms introduce additional parameters and are outside the scope of the present study.

The delayed-feedback mechanism also has a credit-assignment limitation. Because standard RREP messages do not carry the RREQ ID of the discovery that produced them, feedback is matched using only the originator and destination addresses. Overlapping discoveries for the same pair may cause one RREP to update multiple pending decisions, including decisions that did not contribute to that reply. Exact instance-level attribution would require adding an RREQ identifier or another discovery nonce to the reply, which would conflict with the present objective of avoiding packet-format modifications.

The principal comparative evaluation uses a fixed 4-second reward window. Although the sensitivity analysis evaluates W∈{2,4,6,8} s, it is limited to two representative scenarios and does not establish a universally optimal feedback-window duration. The most appropriate redundancy penalty and feedback-window duration may depend on network density, route-discovery timescale, and mobility. Scenarios with substantially different route-discovery timescales may therefore require retuning or online parameter adjustment.

Finally, the traffic workload uses fixed-size CBR UDP flows, and the evaluation is simulation-based. Mixed packet sizes, bursty traffic, heterogeneous application workloads, real interference, and hardware platform constraints are not examined here. These boundaries define the scope of the contribution and motivate future work on adversarial behaviour, mixed workloads, nonstationary mobility, and testbed validation.

## 5. Results and Discussion

### 5.1. Overall Performance

**Figure 1:**
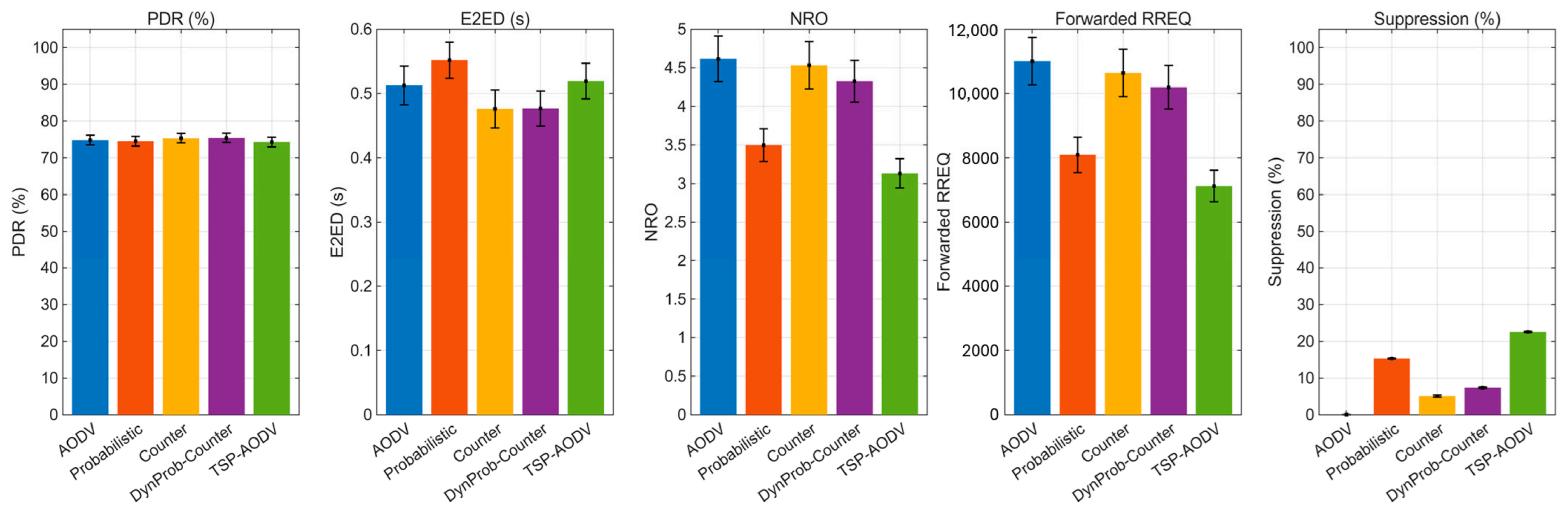
Overall mean performance across the 1600 principal simulation runs. Lower values are better for E2ED, NRO, and RREQ Fwd, whereas higher values are better for PDR and suppression ratio. Error bars show 95% confidence intervals.

Figure 1 provides a compact comparison of all five variants across the key metrics. TSP-AODV achieves the lowest NRO (3.131) and RREQ forwarded count (7119) and the highest suppression ratio (0.226), at a modest PDR cost relative to the counter-based baselines. Table 4 summarises the overall results across all densities, mobility levels, and repetitions.

TSP-AODV achieves the lowest normalised routing overhead, the lowest number of forwarded RREQs, and the highest suppression ratio. This confirms that the proposed method is the most aggressive overhead-reduction mechanism among the evaluated variants.

However, it is not the strongest delivery-oriented method. Counter and DynProbCounter achieve slightly higher PDR and lower delay. Therefore, TSP-AODV should not be interpreted as universally superior. Its main advantage is the overhead–delay trade-off relative to fixed probabilistic rebroadcasting.

### 5.2. Packet Delivery Ratio

**Figure 2:**
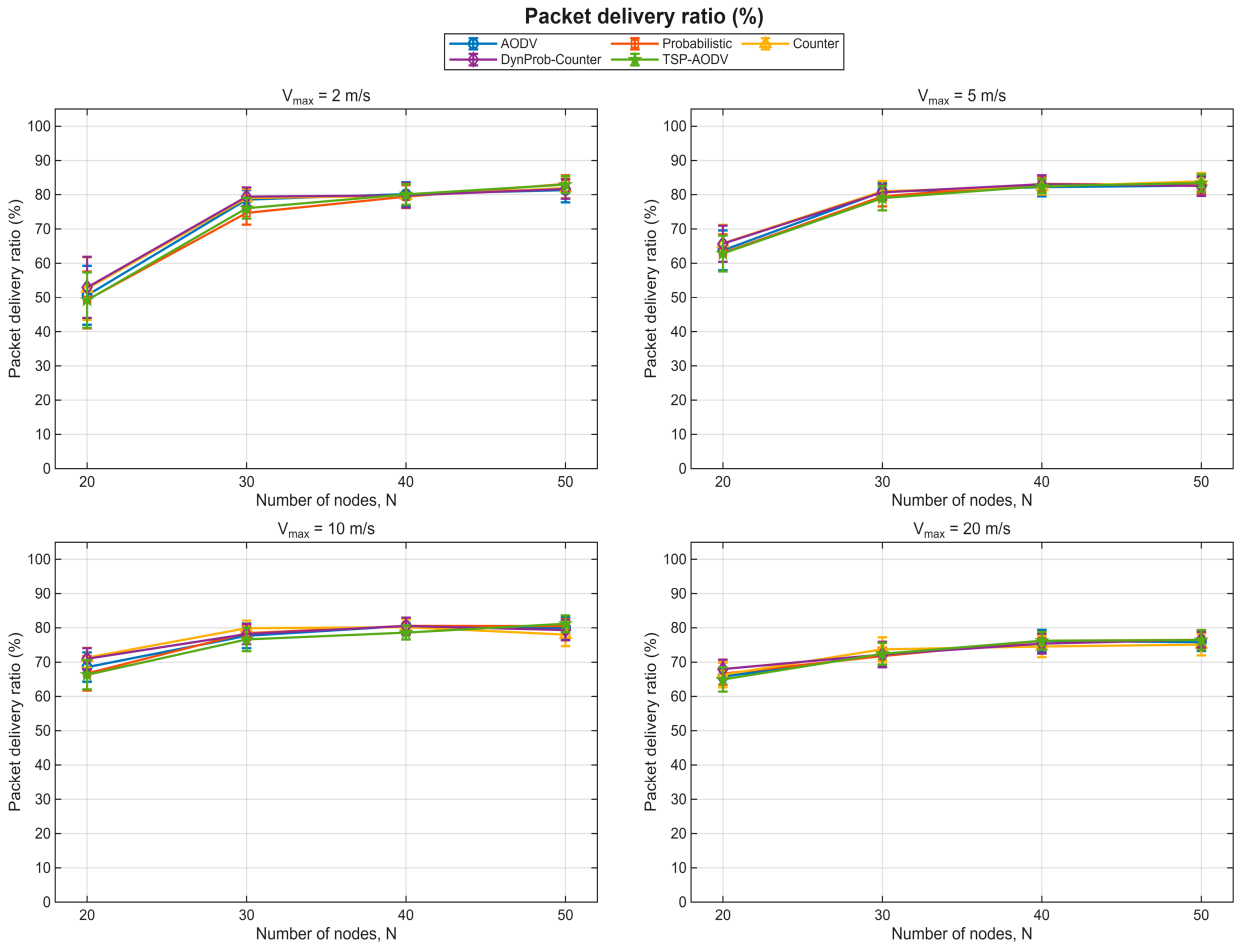
Mean packet delivery ratio (PDR) versus number of nodes, for each maximum speed level. Error bars show 95% confidence intervals over 20 repetitions.

Figure 2 shows PDR as a function of node count for each maximum speed. All variants remain within a relatively narrow range across the 16 density–mobility conditions. TSP-AODV is closest to the probabilistic baseline at all conditions. The pooled paired test yielded p=0.883; therefore, no statistically significant PDR difference was observed under the tested conditions. This result does not establish formal equivalence between TSP-AODV and fixed probabilistic AODV. At N=20, $V_{max}=\;2\;m/s$, which is the sparsest, lowest-mobility scenario, TSP-AODV achieves PDR = 0.492, close to the value obtained by fixed probabilistic (0.493) but below the more conservative Counter (0.526) and DynProbCounter (0.530). At N = 50, $V_{max}\;=\;5\;m/s$, TSP-AODV reaches PDR = 0.834, matching or exceeding all baselines.

### 5.3. End-to-End Delay

**Figure 3:**
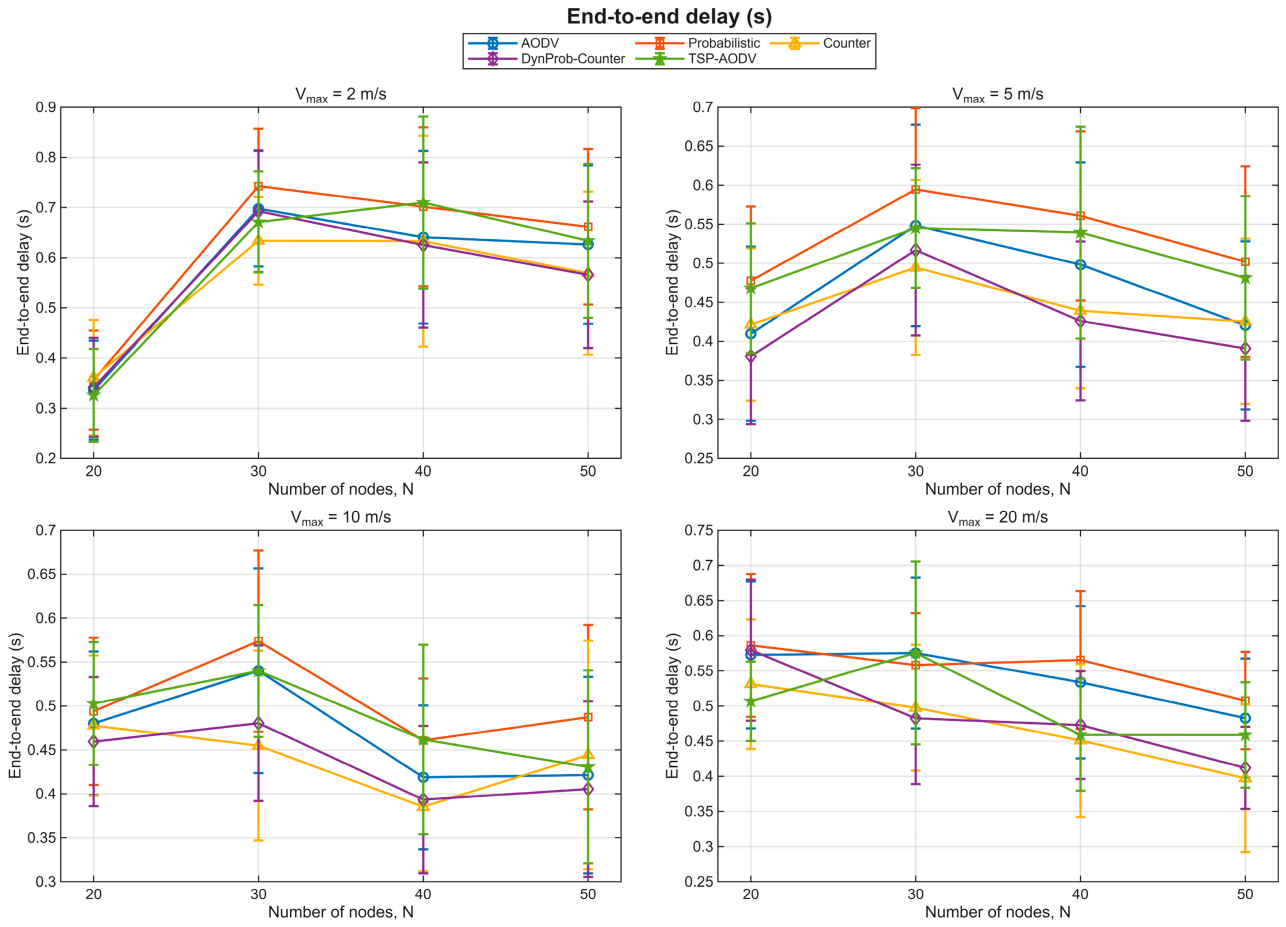
Mean end-to-end delay (s) versus number of nodes for each maximum speed. Error bars show 95% confidence intervals.

Figure 3 shows E2ED results. TSP-AODV significantly reduces delay by 5.96% relative to fixed probabilistic AODV (p < 0.001), driven by reduced channel contention from fewer RREQ transmissions. However, Counter and DynProbCounter achieve lower overall delays of 0.476 s and 0.477 s, respectively, compared with TSP-AODV (0.519 s), because their conservative suppression preserves more forwarding paths and reduces route-discovery latency. TSP-AODV suppresses more broadly, including occasional exploration choices at low and medium duplicate pressure, which can increase latency in borderline connectivity scenarios. This confirms the trade-off introduced in Section 3.3: the assessment interval and exploratory probability selection can reduce contention overall but may also add latency in borderline connectivity conditions. Thus, the delay result should be interpreted as a consequence of two competing effects: fewer RREQ transmissions reduce contention, while assessment waiting and exploratory suppression can increase route-discovery latency in some scenarios.

### 5.4. Normalised Routing Overhead

**Figure 4:**
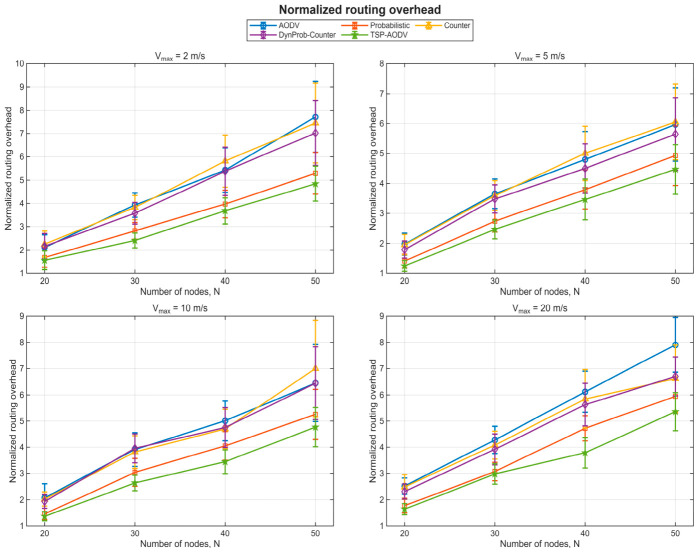
Normalised routing overhead (NRO = AODV control packets/received application packets) versus number of nodes for each maximum speed. Lower values are better. Error bars show 95% confidence intervals over 20 repetitions.

NRO is the metric where TSP-AODV most clearly distinguishes itself (Figure 4). It consistently achieves the lowest NRO across all 16 density–mobility conditions. The gap widens with density: at N = 20, NRO averages 1.452 for TSP-AODV versus 1.581 for fixed probabilistic. At N = 50, the values are 4.861 versus 5.356, which reveals an absolute advantage of nearly 0.5 control packets per delivered data packet. The NRO reduction relative to DynProbCounter is even larger: 27.64% overall (from 4.327 to 3.131, p < 0.001), confirming that learned probability selection achieves substantially more efficient suppression than a rule-based adaptation curve. 

### 5.5. RREQ Forwarding and Suppression Ratio

**Figure 5:**
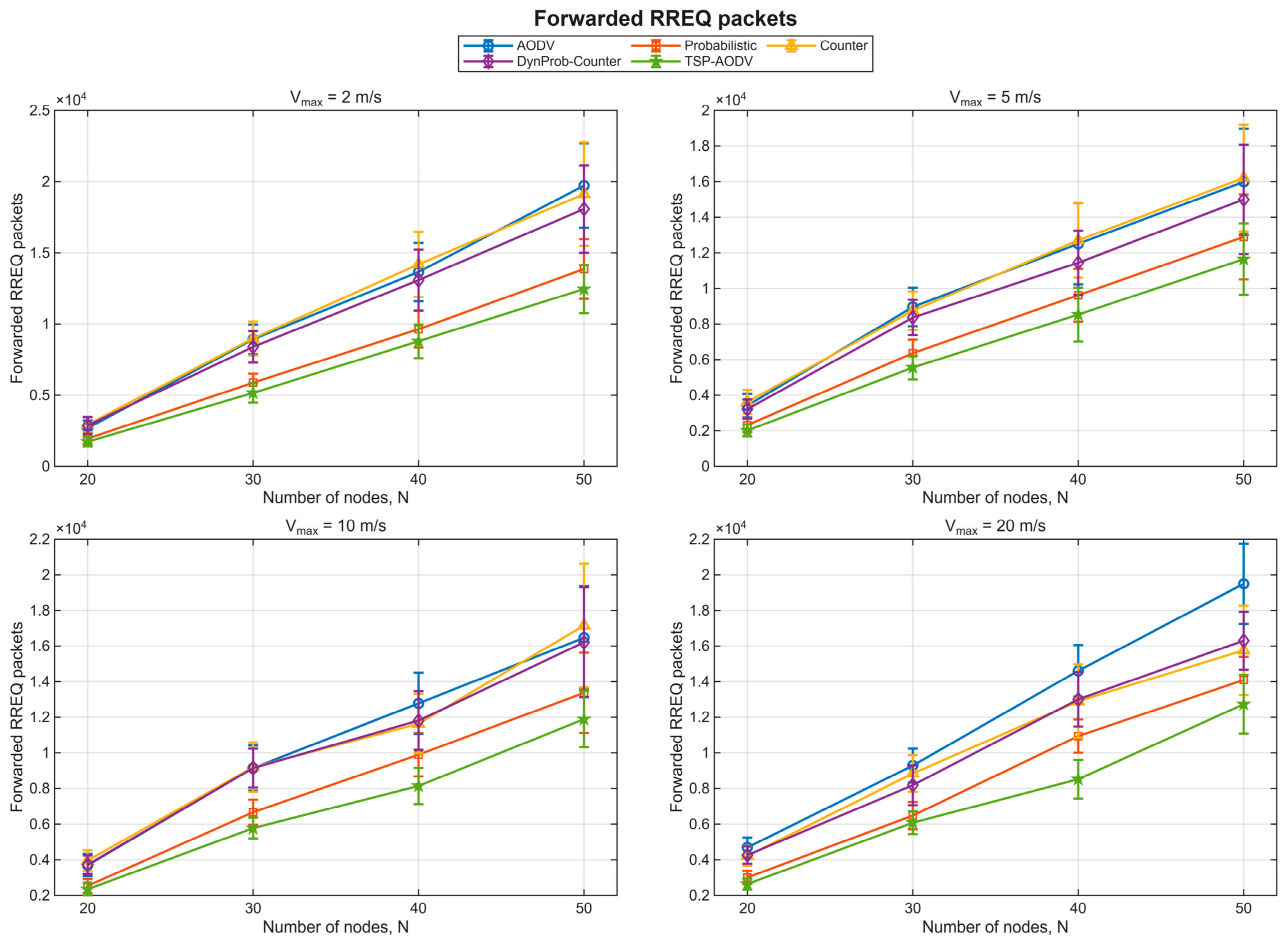
Mean RREQ forwarded count versus number of nodes for each maximum speed. Lower values indicate fewer intermediate-node RREQ rebroadcasts. Error bars show 95% confidence intervals over 20 repetitions.

**Figure 6:**
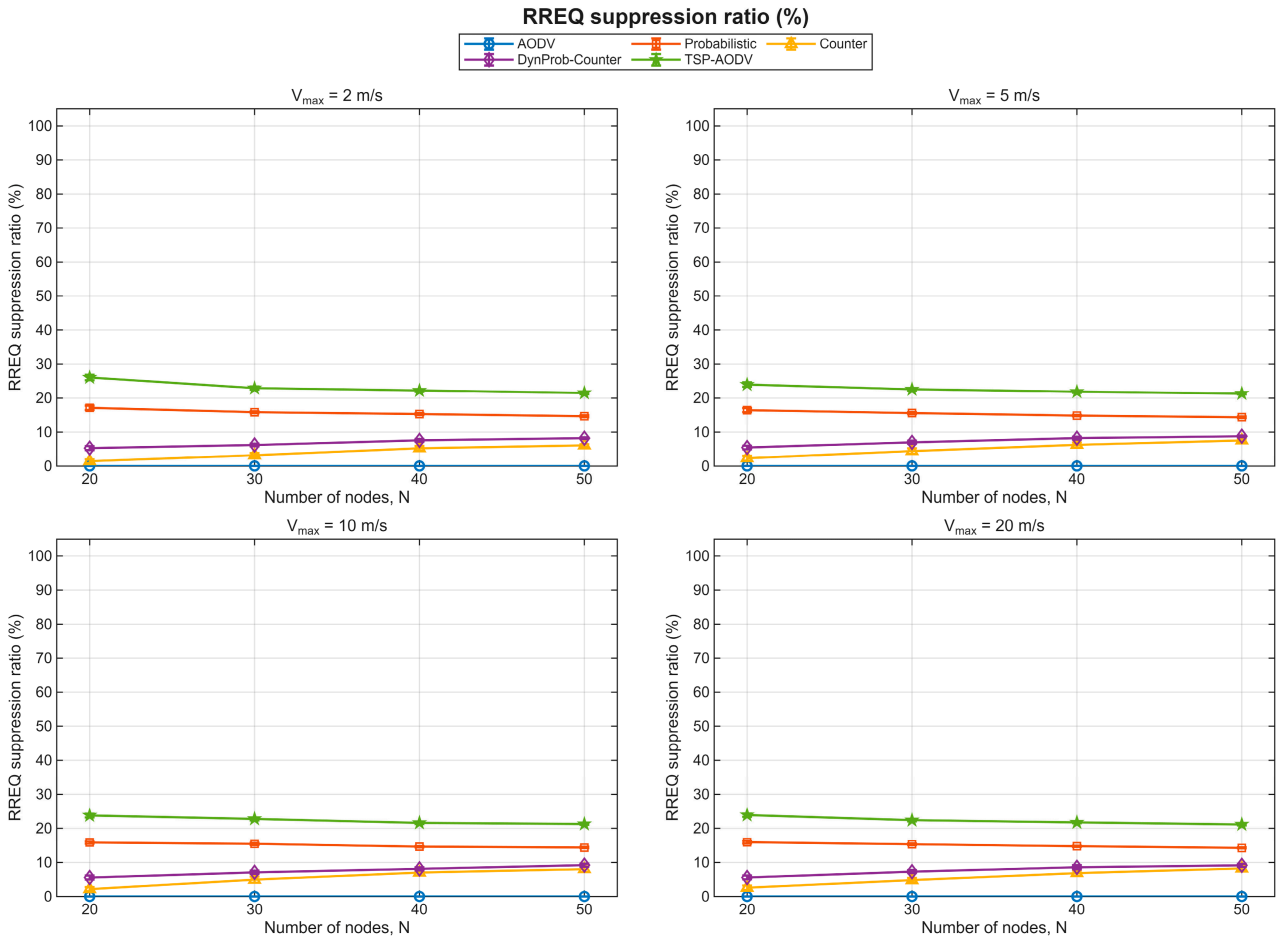
Mean RREQ suppression ratio (suppressed fresh RREQs/received fresh RREQs) versus number of nodes for each maximum speed. Higher values indicate stronger RREQ suppression.

Figure 5 and Figure 6 show RREQ forwarded count and suppression ratio, respectively. TSP-AODV forwards 7119 RREQs on average, versus 8075 for fixed probabilistic (−11.84%,  p < 0.001) and 10,194 for DynProbCounter (−30.16%,  p < 0.001). This reduction comes from a suppression ratio of 0.226, which is the highest of all variants and nearly 3× that of DynProbCounter (0.073). The suppression ratio varies only slightly across the 16 evaluated density–mobility conditions, with a standard deviation of 0.008, indicating consistent suppression behaviour within the tested scenario grid. Counter and DynProbCounter suppress far fewer RREQs (0.051 and 0.073, respectively) because their suppression activates only when the duplicate count exceeds a threshold. At lower densities, many decisions are made without enough duplicates to trigger suppression, whereas TSP-AODV continues to apply probabilistic suppression across all contexts.

### 5.6. Comparison with Fixed Probabilistic AODV

Table 5 reports the paired comparison between TSP-AODV and fixed probabilistic AODV. This is the most important comparison because the proposed method directly extends probabilistic rebroadcasting by replacing the fixed probability p = 0.8 with learned probability selection. To further examine whether these effects are consistent across the evaluated operating conditions, Table 6 reports scenario-level paired *p*-values for each N×Vmax condition. Each cell corresponds to a two-sided paired t-test over the 20 matched repetitions of the corresponding density–mobility scenario.

The exploratory scenario-level results show unadjusted p<0.05 for the suppression-ratio comparison in all 16 conditions and for NRO and RREQ forwarding in many conditions. For PDR, 15 of the 16 unadjusted comparisons have p≥0.05, while one comparison at N=30 and Vmax=2 m/s has p=0.042 and favours TSP-AODV. End-to-end delay has an unadjusted p<0.05 in one condition. Because these 80 secondary tests were not adjusted for multiplicity, no confirmatory inference is drawn from any isolated Table 6 cell. The table is used only to identify where the patterns observed in the pooled analysis appear across the evaluated operating conditions.

### 5.7. Learned Probability Behaviour

**Figure 7:**
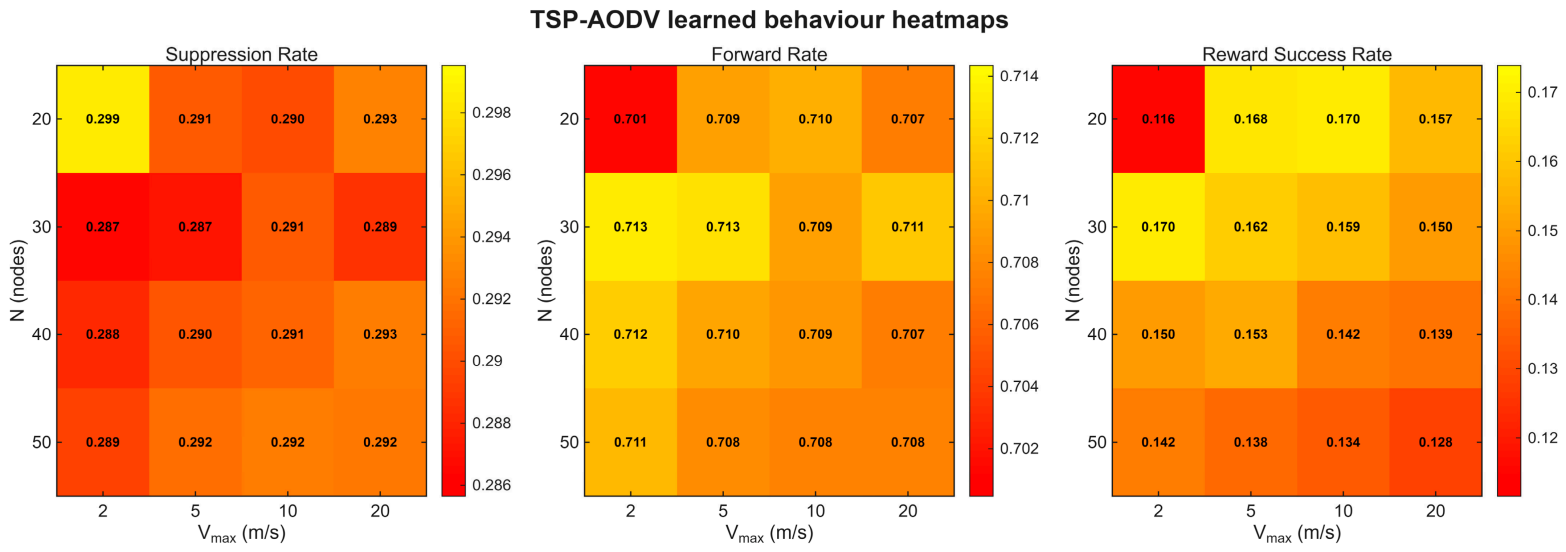
TSP-AODV learned-behaviour heatmaps: TS decision suppression rate, TS forward rate, and reward-success rate by node count (rows) and maximum speed (columns), averaged over 20 repetitions. Cell values show mean rates.

Figure 7 shows TSP-AODV suppression rate, forward rate, and reward success rate across all $16\;N\;\times \;V_{max}$ conditions. The suppression rate (upper panel) varies within a narrow range across the 16 evaluated density–mobility conditions. Table 7 reports the dominant learned arm by duplicate-pressure context.

The policy is monotonically decreasing with duplicate pressure: p = 0.9 under low, p = 0.8 under medium, and p = 0.5 under high pressure. This confirms that TSP-AODV learns a genuinely context-dependent policy rather than converging to a fixed probability. The medium context favouring p = 0.8 rather than p = 0.9 indicates that the belief does differentiate medium from low pressure, though less sharply than it distinguishes high from medium. The reward success rate (mean 0.15) decreasing slightly with density reflects the inherently harder route-discovery conditions in dense, mobile scenarios where collisions and RREP losses are more frequent.

### 5.8. Sensitivity to the Reward Parameters

**Figure 8:**
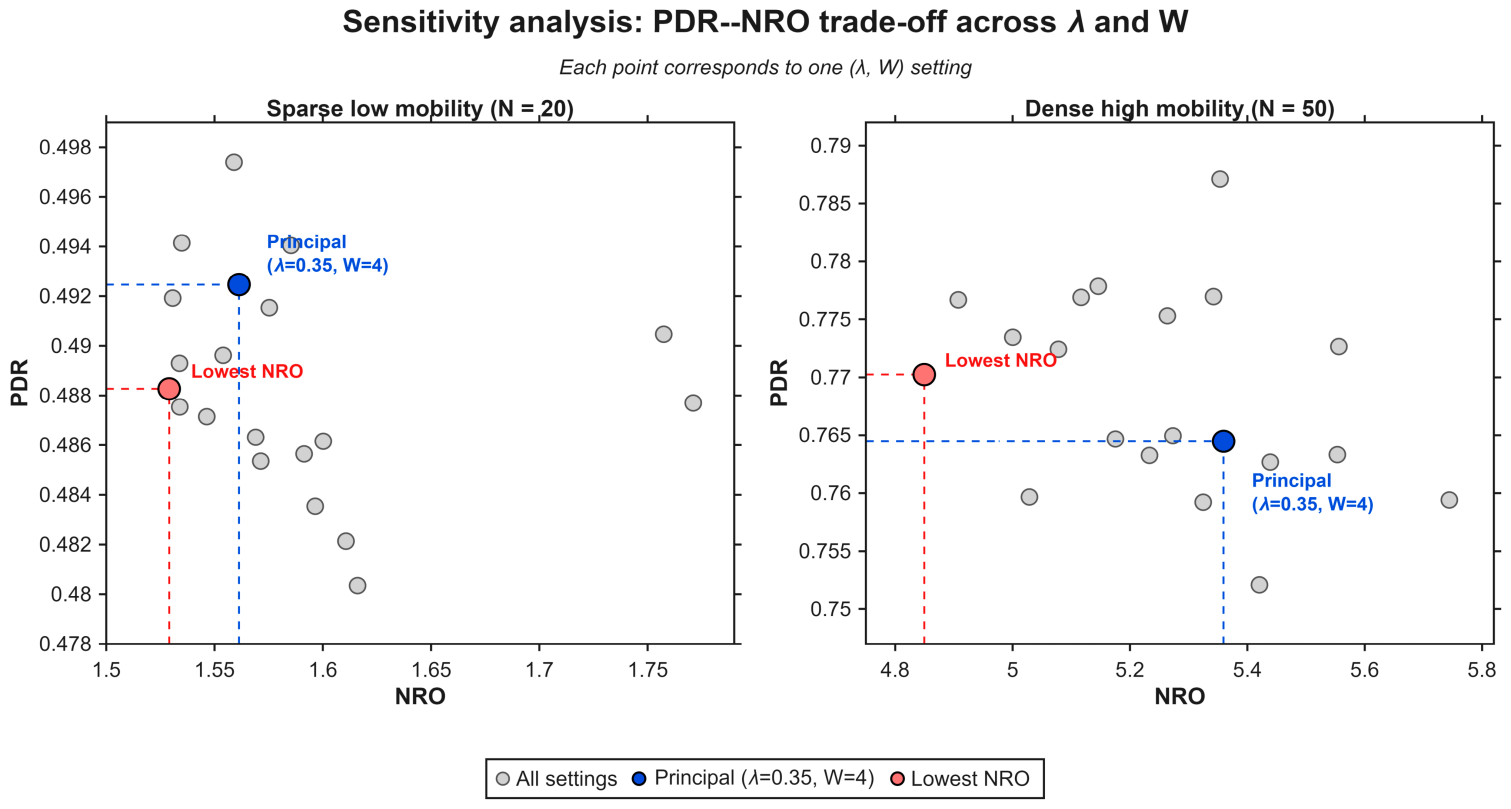
PDR–NRO sensitivity trade-off across the tested (λ, W) settings in sparse low-mobility and dense high-mobility scenarios. Grey points show all tested settings; highlighted points show the principal setting and the lowest NRO setting.

Figure 8 summarises the PDR-NRO trade-off across the tested (λ, W) combinations, with each point representing one reward-parameter setting. In the dense high-mobility scenario, the selected configuration (λ=0.35, W=4) achieves PDR = 0.764, NRO = 5.359, and E2ED=0.459 s. The setting (λ=0.70, W=6) produces more favourable point estimates for all three metrics in this scenario, with PDR = 0.770, NRO = 4.850, and E2ED=0.378 s. In the sparse low-mobility scenario, the selected configuration achieves PDR = 0.492 and NRO = 1.561, whereas the lowest NRO setting (λ=0.50, W=8 s) achieves PDR = 0.488 and NRO = 1.529. 

Table 8 further shows that the lowest NRO and highest PDR settings differ, confirming that the preferred parameter combination depends on whether the objective prioritises delivery, overhead, or delay. Increasing the redundancy penalty may reduce routing overhead by encouraging stronger suppression, but it does not necessarily improve PDR or delay in every evaluated condition. Conversely, parameter settings that improve PDR may produce slightly higher routing overhead. These findings characterise the tested parameter grid in the two selected scenarios and do not support a general claim that TSP-AODV is insensitive to λ or W. The principal setting (λ=0.35, W=4 s) was selected before the sensitivity analysis and retained unchanged across all 16 principal evaluation scenarios to avoid post hoc, scenario-specific tuning. It is reported as a representative common configuration and not as the best parameter choice for every operating condition. Because the sensitivity analysis covers only two scenarios, broader conclusions regarding universal optimality or scenario-independent parameter robustness are not drawn.

### 5.9. Summary

TSP-AODV significantly improves NRO $\left(-10.51\%\right),$ RREQ forwarding $\left(-11.84\%\right),$ end-to-end delay $\left(-5.96\%\right),$ and suppression ratio (+47.48%) relative to fixed probabilistic rebroadcasting. No statistically significant PDR difference was observed under the tested conditions (p=0.883). However, formal equivalence was not evaluated. TSP-AODV is not universally superior, as DynProbCounter and Counter remain more conservative in delivery. The proposed method is therefore best interpreted as an overhead-efficient adaptive probabilistic mechanism whose learned probability preference decreases with increasing duplicate pressure.

## 6. Conclusions

This paper proposed TSP-AODV, a lightweight Thompson-sampling-based extension of probabilistic RREQ suppression for AODV-based mobile ad hoc sensor networks. The method replaces the fixed forwarding probability with online probability selection driven by local duplicate-pressure context and delayed RREP feedback, requiring no additional control packets and maintaining 30 persistent Beta belief parameters per node, supplemented by temporary pending-decision records.

The results show that adaptive probability selection can reduce RREQ overhead relative to fixed probabilistic rebroadcasting. No statistically significant PDR difference was observed under the tested conditions, although formal equivalence was not evaluated. The two-scenario sensitivity analysis identified parameter-dependent trade-offs and showed that the principal setting is not optimal in every evaluated condition. It was retained as a preselected representative configuration for the common evaluation grid, while automatic or scenario-dependent reward-parameter adaptation remains a direction for future work. The method is therefore a lightweight overhead-reduction mechanism for cooperative AODV-based mobile ad hoc sensor networks. 

Future work should examine segmented nonstationary scenarios, selective belief forgetting, adversarial packet-dropping and flooding models, mixed traffic workloads with variable packet sizes and rates, trace-driven or map-constrained mobility, hardware testbed validation, extension to other broadcast-based routing and dissemination protocols, and dynamic reward-window scaling based on TTL or estimated network diameter.

## Figures and Tables

**Table 1:** Classification of representative RREQ/broadcast suppression and learning-based routing approaches.

Work	Category	Main Decision Variable	Adaptation Basis	Relation to TSP-AODV
Tseng et al. [3]	Broadcast storm analysis	Rebroadcast behaviour	Redundancy/contention analysis	Establishes the broadcast storm problem
Williams and Camp [4]	Broadcast suppression taxonomy	Forward/suppress decision	Probabilistic, counter, distance, location, cluster, neighbour knowledge	Classical taxonomy of suppression methods
Haas et al. [14]	Gossip/probabilistic routing	Fixed forwarding probability	Manually selected gossip probability	Shows overhead can be reduced if p is well selected
Reina et al. [15]	Probabilistic broadcast survey	Forwarding probability	Surveyed probabilistic schemes	Highlights sensitivity of fixed probability
Shete and Awale [28]	Adaptive gossip AODV	Forwarding behaviour	Channel-quality rule	Adaptive but rule-based, not TS learning
Cai and Qian [5]	Probabilistic-flooding AODV	RREQ forwarding probability	Multi-constrained fusion	Computes probability through predefined criteria
Tu et al. [6]	Intelligent adaptive AODV	Routing/forwarding indicators	Local network and link-quality indicators	Broader adaptive AODV, not RREQ probability learning
McCarthy et al. [7]	Energy-aware AODV	Route selection/weighting	Energy-optimised combined weighting	Energy-aware routing, not RREQ suppression learning
Safari et al. [8]	Cross-layer fuzzy AODV	Route/forwarding decision	Fuzzy cross-layer metrics	Rule-based/fuzzy adaptation
Baumgartner et al. [9]	Resilient MANET routing	Routing robustness	Protocol resilience mechanisms	Improves robustness, not TS-based RREQ suppression
Levis et al. [37]	Trickle suppression	Broadcast/suppression timing	Consistency and redundancy	Related suppression principle outside AODV route discovery
Meyfroyt et al. [38]	Adaptive Trickle suppression	Redundancy constant	Adaptive suppression parameter	Supports need for adaptive suppression parameters
Coladon et al. [39]	Multiple Trickle constants	Redundancy constant	Multiple redundancy settings	Shows fixed suppression constants can be limiting
Lansky et al. [10]	RL routing survey	Route/next-hop decisions	Reinforcement learning	Broader learning-based routing context
Bouneffouf et al. [11]	Bandit survey	General finite-action decision	MAB/contextual bandit feedback	Supports lightweight bandit formulation
Huang et al. [12]	Thompson-sampling routing	Opportunistic forwarding candidates	TS-based candidate/path selection	Uses TS for forwarding/path choice, not RREQ rebroadcast probability
Riou and Honda [42]	Bounded-reward TS	Bounded reward learning	Thompson sampling for bounded rewards	Supports bounded-feedback interpretation

**Table 2:** Compared RREQ rebroadcast variants.

Variant	Rebroadcast Decision	Adaptive?	Main Parameter(s)
AODV	Forward all fresh RREQs	No	—
Probabilistic	Forward each fresh RREQ with fixed p	No	p=0.8
Counter	Suppress if duplicate count ≥ threshold	Rule-based	C = 3
DynProbCounter	$p\left(D\right)$ decreases with duplicate count	Rule-based	$p_{min}=0.35$,
			$p_{max}=1.0$,
			$D_{ref}$ = 3,
			δ=1.0
TSP-AODV	Thompson sampling over probability arms with duplicate-pressure context	Learned	𝒜 = {0.5, 0.6, 0.7, 0.8, 0.9},
			Principal setting: λ=0.35

**Table 3:** Simulation parameters.

Parameter	Value
Simulator	OMNeT++ 6.3.0/INET 4.5.4
Routing protocol	AODV with modified RREQ rebroadcast decision
Simulation area	1000 m×1000 m
Number of nodes	20, 30, 40, 50
Mobility model	Random Waypoint
Minimum speed	0.1 m/s
Maximum speed	{2, 5, 10, 20} m/s
Pause time	2 s
MAC protocol	IEEE 802.11 MAC, ad hoc mode
Radio model	IEEE 802.11 scalar radio/scalar radio medium
Data rate	2 Mbps
Communication range	250 m
Traffic pattern	CBR over UDP
Number of source–destination flows	10
Packet size	512 bytes
Packet generation interval	0.25 s per source
Packet generation rate	4 packets/s per flow, 40 packets/s aggregate
Simulation duration	125 s
Warm-up period	5 s
Repetitions per scenario	20 per scenario
Total runs	1600 (5 variants × 4 densities × 4 speeds × 20 reps)
Redundancy-penalty coefficient	λ=0.35 in the principal evaluation
Assessment interval $\Delta_{assess}$	uniform (0 ms, 10 ms)
Duplicate reference count $D_{ref}$	3
Feedback window	W=4 s in the principal evaluation
Sensitivity values	$\lambda \in \left\{0,\;0.20,\;0.35,\;0.50,\;0.70\right\},\;W\in \left\{2,\;4,\;6,\;8\right\}\;s\;$
Sensitivity analysis scenarios	N=20, $V_{max}\;=\;2\;m/s$ and N=50, $V_{max}\;=\;20\;m/s$
Repetitions per sensitivity setting	20 matched repetitions
Additional sensitivity runs	800 (5×4×2 scenarios×20 reps)

**Table 4:** Overall mean performance across all simulation runs. Bold indicates the best value per metric.

Variant	PDR	Delay (s)	NRO	RREQ Fwd	Supp. Ratio
AODV	0.748	0.512	4.615	11,003	0.000
Probabilistic	0.744	0.552	3.499	8075	0.153
Counter	0.753	**0.476**	4.533	10,637	0.051
DynProbCounter	**0.754**	0.477	4.327	10,194	0.073
**TSP-AODV**	0.743	0.519	**3.131**	**7119**	**0.226**

**Table 5:** Paired comparison of TSP-AODV versus fixed probabilistic AODV (matched runs across all density, mobility, and repetition combinations, two-sided paired *t*-test).

Metric	TSP-AODV	Probabilistic	Change	Significance
PDR	0.743	0.744	−0.07%	Not significant (*p* = 0.883)
End-to-end delay (s)	0.519	0.552	−5.96%	Significant (*p* < 0.001)
NRO (ctrl/delivered)	3.131	3.499	−10.51%	Significant (*p* < 0.001)
RREQ forwarded	7119	8075	−11.84%	Significant (*p* < 0.001)
Suppression ratio	0.226	0.153	+47.48%	Significant (*p* < 0.001)

**Table 6:** Exploratory scenario-level paired *p*-values for TSP-AODV versus fixed probabilistic AODV for each N×Vmax condition. Each cell reports an unadjusted two-sided paired-test *p*-value over 20 matched repetitions. The table contains 80 secondary comparisons and is used only to localise patterns observed in the pooled analysis. No confirmatory inference is drawn from an isolated cell.

Metric	N	$V_{max}=2$	$V_{max}=5$	$V_{max}=10$	$V_{max}=20$
PDR	20	0.908	0.858	0.803	0.461
	30	0.042	0.537	0.252	0.690
	40	0.296	0.307	0.181	0.265
	50	0.743	0.565	0.557	0.967
E2ED (s)	20	0.218	0.781	0.819	0.172
	30	0.137	0.113	0.236	0.686
	40	0.859	0.513	0.982	0.025
	50	0.174	0.509	0.054	0.293
NRO	20	<0.001	0.005	0.143	0.389
	30	0.004	0.023	0.042	0.616
	40	0.001	0.008	0.007	0.002
	50	0.007	0.002	0.127	0.199
RREQ forwarded	20	<0.001	0.001	0.074	0.059
	30	0.001	0.004	<0.001	0.267
	40	<0.001	<0.001	0.003	0.002
	50	0.003	0.001	0.052	0.195
Suppression ratio	20	<0.001	<0.001	<0.001	<0.001
	30	<0.001	<0.001	<0.001	<0.001
	40	<0.001	<0.001	<0.001	<0.001
	50	<0.001	<0.001	<0.001	<0.001

**Table 7:** Dominant learned forwarding probability by duplicate-pressure context (final belief means).

Duplicate-Pressure Context	Dominant Learned Arm
Low	*p* = 0.9
Medium	*p* = 0.8
High	*p* = 0.5

**Table 8:** Best and selected sensitivity settings in the sparse and dense representative scenarios.

Scenario	Setting	λ	W	PDR	NRO	E2ED (s)	Suppression
Dense/high mobility	Selected/main	0.35	4	0.764	5.359	0.459	0.292
Dense/high mobility	Lowest NRO	0.70	6	0.770	4.850	0.378	0.296
Dense/high mobility	Highest PDR	0.35	2	0.787	5.353	0.415	0.289
Sparse/low mobility	Selected/main	0.35	4	0.492	1.561	0.326	0.299
Sparse/low mobility	Lowest NRO	0.50	8	0.488	1.529	0.414	0.298
Sparse/low mobility	Highest PDR	0.50	4	0.497	1.559	0.350	0.295

## Data Availability

The processed simulation data supporting the reported statistical analysis are available from the corresponding author upon reasonable request. The source code is not publicly released.

## Abbreviations

The following abbreviations are used in this manuscript:

AODV	Ad hoc on-demand distance vector
CBR	Constant bit rate
E2ED	End-to-end delay
IoT	Internet of Things
MAC	Medium access control
MANET	Mobile ad hoc network
NRO	Normalised routing overhead
PDR	Packet delivery ratio
RREP	Route reply
RREQ	Route request
RL	Reinforcement learning
RWP	Random waypoint
TS	Thompson sampling
TSP-AODV	Thompson sampling probabilistic AODV
UDP	User Datagram Protocol
